# Phosphorus Containing Polyacrylamides as Flame Retardants for Epoxy-Based Composites in Aviation

**DOI:** 10.3390/polym11020284

**Published:** 2019-02-08

**Authors:** Lara Greiner, Philipp Kukla, Sebastian Eibl, Manfred Döring

**Affiliations:** 1 Fraunhofer Institute for Structural Durability and System Reliability LBF, Schlossgartenstr. 6, 64289 Darmstadt, Germany; lara.greiner@lbf.fraunhofer.de (L.G.); philipp.kukla@lbf.fraunhofer.de (P.K.); 2 Bundeswehr Research Institute for Materials, Fuels and Lubricants, Institutsweg 1, 85435 Erding, Germany; SebastianEibl@bundeswehr.org

**Keywords:** epoxy resin, composite, fiber degradation, phosphorus-containing flame retardants

## Abstract

Novel polymeric flame retardants based on two acrylamides and 9,10-dihydro-9-oxa-10-phosphaphenanthrene-10-oxide (DOPO) or 5,5-dimethyl-[1,3,2]dioxaphosphinane-2-oxide (DDPO) are described for several applications in HexFlow^®^ RTM6, a high-performance epoxy resin. Neat resin samples and carbon fiber-reinforced composites were tested for their glass transition temperatures (dynamic mechanical analysis), thermal stability (thermogravimetric analyses), flammability (UL94) and flame-retardant performance (Cone Calorimetry). Additionally, the fiber degradation occurring during combustion of carbon fiber-reinforced epoxy resins was observed by scanning electron microscopy to show the fiber protecting effect of these flame retardants. Whereas DOPO-containing polyacrylamides acting mainly in the gas phase showed the best flame retardant efficiency, DDPO-containing polyacrylamides acting mainly in the condensed phase showed the best fiber protection. A mixed polyacrylamide was synthesized to combine these effects. This thermoplastic is soluble in the resin and, therefore, suitable for injection molding processes. Interlaminar shear strength measurements showed no negative effect of the flame retardant. The versatility of these flame retardants is shown by investigations dealing with boehmite as synergist in neat resin samples.

## 1. Introduction

Epoxy-based composites can be found in numerous highly sophisticated aerospace applications. In particular, carbon fiber reinforcement leads to lightweight materials with optimized mechanical properties. For many applications, adjusted flame retardancy is necessary. The efficiency of incorporated flame retardants depends on the epoxy resin matrix and the reinforcement [1,2,3,4,5,6,7,8,9,10].

Moreover, it is well known that carbon fibers can form respirable fiber fragments after a thermal load and fire [11,12]. Several aircraft crashes accompanied by fire have increased the interest in assessing the additional health risk of respirable carbon fiber dust. Critical fiber concentrations were reported for large-scale fire tests and collecting the flight recorder of a crashed helicopter [13,14]. Carbon fibers consist of more than 90% carbon (typically over 95%) with a residue of mainly nitrogen, which makes them prone to oxidation reactions. Within minutes a significant oxidation reaction is observed above 600 °C in air. This reaction is influenced by heating rate and strongly influenced by oxygen concentration [15]. A continuous decrease of fiber diameter and a formation of surface defects, which end up in a pronounced hole structure at severe damage, have been observed. After 10 min at 650 °C, fibers thinner than 3 µm were detected [15]. According to the definition of the World Health Organization (WHO), fibers thinner than 3 µm longer than 5 µm are considered to be respirable. Additionally, the length to diameter ratio has to be higher than 3. Fibers with these dimensions are thin enough to penetrate the deep lung areas (alveoli), and too long to be exhaled [16]. As long as carbon fiber-reinforced thermoset polymers (CFRP) are not exposed to high temperatures or other degradation processes, commercially available carbon fibers are typically thicker than 5 µm and, therefore, uncritical with regard to inhalation. Consequently there is a need for fiber protection in addition to flame retardancy. With respect to fiber-matrix adhesion, literature [15,17] showed that the addition of zinc borate shows fiber protection by the formation of a glassy surface layer on the fibers during matrix combustion. However, zinc borate is not suitable for resin transfer molding, an important process for the manufacturing of fiber-reinforced epoxy based composites. It increases the viscosity of the resin significantly and the inhomogeneously dispersed particles are filtered by the fiber plies during injection.

Widely discussed flame retardants for epoxy resins and composites thereof are phosphorus containing compounds. For phosphorus containing flame retardants, the mode of action depends on the phosphorus species and the polymer they are incorporated [18,19]. If the chemical environment of the phosphorus is carbon/hydrogen rich, it promotes action in the gas phase. An oxygen rich environment promotes action in the condensed phase, respectively. Moreover, a trend to polymeric, oligomeric or star-shaped flame retardants is observed to avoid leaching and to ensure the high mechanical and thermal requirements of the composites [1,2,9,20,21,22,23,24,25,26]. Investigations with triazines [27] or phosphazenes [28] showed the synergistic effects of phosphorus and nitrogen containing units.

**Figure 1:**
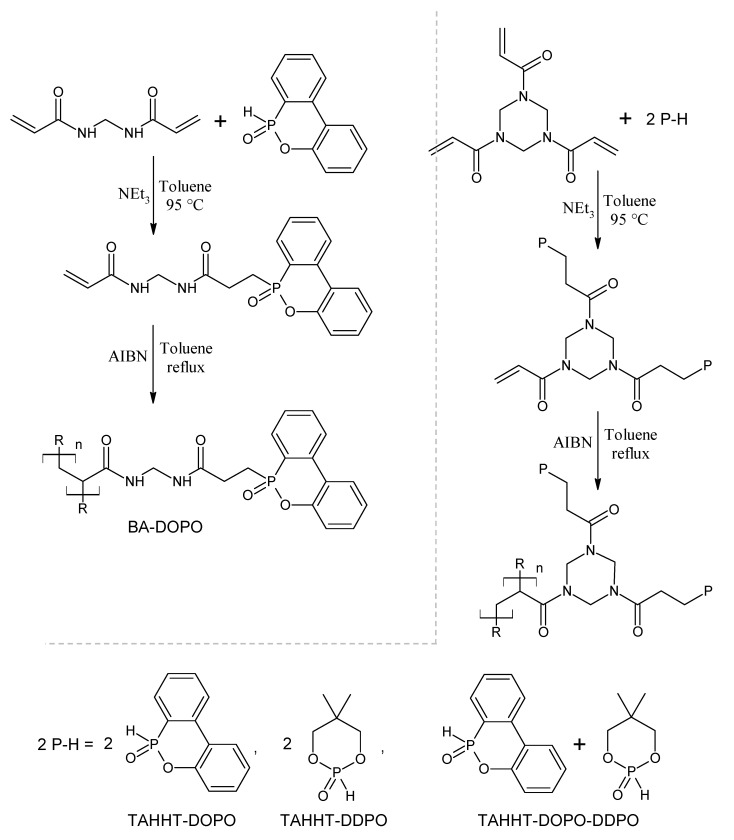
Synthesis of BA-DOPO, TAHHT-DOPO, TAHHT-DDPO and TAHHT-DOPO-DDPO. Detailed ratios are shown in the synthesis descriptions.

To combine different phosphorus species in polymeric or oligomeric nitrogen-containing molecules, a new approach is investigated. Oligomeric flame retardants were synthesized by Phospha-Michael addition of phosphorus compounds like the carbon rich 9,10-dihydro-9-oxa-10-phosphaphenanthrene-10-oxide (DOPO) or the oxygen rich 5,5-dimethyl-1,3,2-dioxaphosphorinan-2-one (DDPO) and multifunctional acrylamides with the following radical polymerization of residual double bonds (Figure 1). Dependent on the acrylamide used (e.g. *N*,*N*′-methylenebisacrylamide (BA) or 1,3,5-triacryloylhexahydro-1,3,5-triazine (TAHHT)) and the ratio of phosphorus containing molecules to double bonds, oligomeric or polymeric products with either thermoset or thermoplastic properties are formed. Whereas DOPO, which is more likely to act in the gas-phase, shall provide mainly flame retardancy for carbon fiber-reinforced epoxy resins, DDPO may initiate the formation a protective layer on the carbon fibers to retard the formation of respirable carbon fiber fragments. This work compares flame retardant properties and fiber protection of BA-DOPO, TAHHT-DOPO and TAHHT-DOPO-DDPO with thermoplastic properties and TAHHT-DDPO with thermoset properties. Due to the lower reactivity of the P-H-group in DDPO, the conversion of double bonds by Phospha-Michael addition was not sufficient to yield a thermoplastic compound.

The following work includes an additional possible application for the novel flame retardants in a prepreg system matrix. Aluminum hydroxides, especially aluminum tri-hydroxide (ATH), are often used as flame retardant inorganic fillers. Due to low flame retardant efficiencies, high loadings of the hydroxides are required and have a negative effect on mechanical properties and processability [29]. Therefore, the filler loading is reduced by addition of synergistic flame retardants like phosphorus-based compounds [30,31]. For epoxy resins, there are known synergists in combination with ATH. Boehmite is an inorganic filler used for temperatures up to 300 °C. The synergistic effect of boehmite together with DOPO is described in the literature for epoxy resins like bisphenol A diglycidyl ether hardened with dicyandiamide and fenuron [30]. A lower surface area of boehmite results in more flame retardant species in combination with phosphinates [3]. 

These investigations show the synergistic effect of the novel phosphorus containing polyacrylamides and two different types of boehmite in a high-performance epoxy resin.

## 2. Materials and Methods

Unless stated otherwise, solvents and chemicals were obtained from commercial sources and used as received.

### 2.1. Synthesis of BA-DOPO

Into a 1 L three-necked round bottom flask equipped with a thermometer, a reflux condenser with a drying tube (sodium hydroxide filled) and a sealed precision glass stirrer 600 mL toluene, 18 mL (130 mmol) triethylamine (anhydrous), 200 mg 4-methoxyphenol and 50.0 g (324 mmol, 1.00 eq.) *N*,*N*′-methylenebisacrylamide were introduced. The mixture was heated to 95 °C while stirring and under vigorous stirring, 73.5 g (340 mmol, 1.05 eq.) DOPO was added in small portions within 3 h. After continuing stirring at 95 °C for additional 25 h, the full conversion of DOPO was observed by ^31^P-nuclear magnetic resonance (NMR) (CDCl_3_, 300 MHz, 298 K, NanoBay 300, Bruker, Billerica, MA, USA): 37.64 ppm (m). The drying tube was exchanged with a nitrogen inlet and the apparatus was filled with nitrogen. The mixture was refluxed for 1 h. Afterwards, 1.5 mL of 2,2′-azobis(2-methylpropionitrile) (AIBN) solved in toluene (0.2 mol·L^−1^) was added. After 5 min another 0.5 mL of this solution was added. The mixture was refluxed for additional 3 h during which a viscous oligomer precipitated. After cooling down to about 60 °C, the solution was filtered and the product was washed with toluene. Drying in vacuo at 90 °C for 24 h resulted in a white powder. The yield was 118 g (96%). ^31^P-NMR (CDCl_3_, 300 MHz, 298 K): 37.25 ppm (m); ^1^H-NMR (CDCl_3_, 300 MHz, 298 K): 7.92 ppm (NH, m, 1H), 7.89 ppm (NH, m, 1H) 7.69 ppm (CH_arom_, m, 1H), 7.50 ppm (CH_arom_, m, 1H), 7.36 ppm (CH_arom_, m, 1H), 7.18 ppm (CH_arom_, m, 4H), 6.96 ppm (CH_arom_, m, 1H), 4.63 ppm + 4.51 ppm (CH_2_, t, ^3^J_HH_ 6.12 Hz + 5.77 Hz, 2H), 2.49 ppm + 2.39 ppm (CH_Backbone_/CH_2_, m/s, 7H).

### 2.2. Synthesis of TAHHT-DOPO

Into a 1 L three-necked round bottom flask equipped with a thermometer, a reflux condenser with a drying tube (sodium hydroxide filled) and a sealed precision glass stirrer 400 mL toluene, 12 mL (87 mmol) triethylamine (anhydrous), 200 mg 4-methoxyphenol and 30.0 g (120 mmol, 1.00 eq.) 1,3,5-triacryloylhexahydro-1,3,5-triazine were introduced. The mixture was heated to 95 °C while stirring and under vigorous stirring, 54.4 g (252 mmol, 2.10 eq.) DOPO was added in small portions within 3 h. After continuing stirring at 95 °C for additional 15 h, the full conversion of DOPO was observed by ^31^P-NMR (CDCl_3_, 300 MHz, 298 K): 37.34 ppm (m). The drying tube was exchanged by a nitrogen inlet and the apparatus was filled with nitrogen. The mixture was refluxed for 2 h. Afterwards 1.2 mL of 2,2′-azobis(2-methylpropionitrile) solved in toluene (0.2 mol·L^−1^) was added. After 5 min another 1.0 mL of this solution were added. The mixture was refluxed for additional 3 h during which a viscous oligomer precipitated. After cooling down to about 60 °C, the solution was filtered and the product was washed with toluene. Drying in vacuo at 130 °C for 12 h resulted in a white powder. The yield was 83 g (98%). ^31^P-NMR (CDCl_3_, 300 MHz, 298 K): 37.27 ppm (m); ^1^H-NMR (CDCl_3_, 300 MHz, 298 K): 7.89 ppm (CH_arom_, m, 4H), 7.64 ppm (CH_arom_, m, 2H), 7.46 ppm (CH_arom_, m, 2H), 7.18 ppm (CH_arom_, m, 8H), 5.19 ppm (C_aliph_, m, 6H), 2.84 ppm (C_Backbone_, m, 3H), 2.35 ppm (C_aliph_, s, 8H).

### 2.3. Synthesis of TAHHT-DDPO

Into a 1 L three-necked round bottom flask equipped with a thermometer, a reflux condenser with a drying tube (sodium hydroxide filled) and a sealed precision glass stirrer 400 mL toluene, 12 mL (87 mmol) triethylamine (anhydrous), 200 mg 4-methoxyphenol and 30.0 g (120 mmol, 1.00 eq.) 1,3,5-triacryloylhexahydro-1,3,5-triazine were introduced. The mixture was heated to 95 °C while stirring and under vigorous stirring, 32.0 g (216 mmol, 1.80 eq.) DDPO was added in small portions within 3 h. After continuing stirring at 95 °C for additional 15 h, the full conversion of DDPO was observed by ^31^P-NMR (CDCl_3_, 300 MHz, 298 K): 27.38 ppm (m). The drying tube was exchanged by a nitrogen inlet and the apparatus was filled with nitrogen. The mixture was refluxed for 2 h. Afterwards 1.2 mL of 2,2′-azobis(2-methylpropionitrile) solved in toluene (0.2 mol·L^−1^) was added. After 5 min another 1.0 mL of this solution were added. The mixture was refluxed for additional 3 h during which a viscous oligomer precipitated. After cooling down to about 60 °C, the solution was filtered and the product was washed with toluene. Drying in vacuo at 110 °C for 15 h resulted in a white insoluble powder with thermoset properties. The yield was 57 g (92%). 

### 2.4. Synthesis of TAHHT-DOPO-DDPO

Into a 1 L three-necked round bottom flask equipped with a thermometer, a reflux condenser with a drying tube (sodium hydroxide filled) and a sealed precision glass stirrer 400 mL toluene, 12 mL (87 mmol) triethylamine (anhydrous), 200 mg 4-methoxyphenol and 30.0 g (120 mmol, 1.00 eq.) 1,3,5-triacryloylhexahydro-1,3,5-triazine were introduced. The mixture was heated to 95 °C while stirring and under vigorous stirring, 17.8 g (120 mmol, 1.00 eq.) DDPO was added in small portions within 3 h. After continuing stirring at 95 °C for 25 h, 28.5 g (132 mmol, 1.10 eq) DOPO were added in small portions within 2 h. After another 10 h at 95 °C, the full conversion of DOPO and DDPO was observed by ^31^P-NMR (CDCl_3_, 300 MHz, 298 K): 27.44 ppm (m), 37,29 ppm (s). The drying tube was exchanged by a nitrogen inlet and the apparatus was filled with nitrogen. The mixture was refluxed for 2 h. Afterwards 1.2 mL of 2,2′-azobis(2-methylpropionitrile) solved in toluene (0.2 mol·L^−1^) was added. After 5 min, another 1.0 mL of this solution were added. The mixture was refluxed for additional 3 h during which a viscous oligomer precipitated. After cooling down to about 60 °C, the solution was filtered and the product was washed with toluene. Drying in vacuo at 110 °C for 15 h resulted in a white powder. The yield was 74 g (97%). ^31^P-NMR (CDCl_3_, 300 MHz, 298 K): 27.33 ppm (m, DDPO, 0.45 equivalent), 37.31 ppm (s, DOPO, 0.55 equivalent).

### 2.5. Characterization

Pure flame retardants were analyzed by thermogravimetric analyses (TGA; Q500, TA Instruments, Inc., New Castle, DE, USA) starting at room temperature and heated to 800 °C under nitrogen or synthetic air (50 ml/min) with a heating rate of 10 K·min^−1^.

Reaction-to-fire tests were performed with the carbon fiber reinforced system HexFlow^®^ RTM6 (aromatic epoxy resin matrix) and HexForce^®^ G0939 (carbon fiber fabric) from Hexcel Composites GmbH (Stade, Germany) [32]. The matrix was modified by amounts of 10% by matrix weight of BA-DOPO, TAHHT-DOPO, TAHHT-DDPO or TAHHT-DOPO-DDPO. Mixing was carried out at 120 °C for 15 min. Eight plies were laminated by hand, resulting in 2 mm thick lay-ups: [(0|90)]_8_. This procedure is described in the literature [33]. The specimens were cured in an autoclave according to the manufacturer’s recommended conditions [32]. The cured laminates were cut into specimens of 100 × 100 mm for cone calorimetric measurements and specimens of 20 × 10 mm for the measurement of the interlaminar shear strength with a water-cooled diamond wheel saw. Additionally, samples of flame retarded RTM6 without fiber reinforcement were prepared (2 mm, 4 mm). Curing of these samples was carried out with the same temperature program as the composites but under atmospheric pressure. Specimens of 30 × 10 × 2 mm for dynamic mechanical analysis, 70 × 13 mm × 4 mm for UL94-tests were trimmed with a band-saw. 

Synergism with boehmite was tested with synthetic boehmite Actilox^®^ B30 (Nabaltec AG, Schwandorf, Germany) and Disperal^®^ 40 (Sasol Germany GmbH, Brunsbüttel, Germany). Actilox^®^ B30 is a high purity boehmite (99%) with a particle size D_50_ of 1.8 µm and specific surface area (BET) of 3 m²·g^−1^. Disperal^®^ 40 is an acid dispersible boehmite with lower purity (94%) and bigger particles (35 µm) with higher BET-surface (100 m²·g^−1^). Neat resin samples were prepared as described above for 35% boehmite and 30% boehmite in combination with 5% TAHHT-DDPO or TAHHT-DOPO.

An overview of the prepared samples is given in Table 1.

Specimens 4 mm thick were analyzed by UL-94 (Dr.-Ing. Georg Wazau Mess- + Prüfsysteme GmbH, Berlin, Germany) according to DIN EN 60695-11-10 [34] and 2 mm thick specimens were investigated by dynamic mechanical analysis (DMA; SEIKO SII EXSTAR 6100 DMS, Seiko Instruments Inc., Chiba, Japan) with single cantilever geometry from 35 °C to 260 °C with a frequency of 1 Hz and a heating rate of 3 K·min^−1^. Thermogravimetric analyses (TGA; Q500, TA Instruments, Inc., New Castle, DE, USA) of the compact material were performed starting at room temperature and heated to 800 °C under nitrogen or synthetic air (50 ml/min) with a heating rate of 10 K·min^−1^.

Cone calorimetric measurements (Fire Testing Technology Ltd, East Grinstead, England) of fiber-reinforced specimens were carried out in order to determine reaction-to-fire characteristics such as heat release, mass loss, formation of smoke etc. [35] according to ISO 5660 [36] in non-scrubbed mode. Specimens (100 × 100 mm^2^) were wrapped in aluminum foil at the backside, supported on mineral wool, inserted in a frame sample holder and irradiated at a heat flux of 60 kW·m^−2^ for reinforced samples and at a heat flux of 35 kW·m^−2^ for neat resin samples. 60 kW·m^−2^ correspond more to fully developed fires and are used to observe the fiber degradation while 35 kW·m^−2^ correspond to developing fires [37]. Test duration (5 min, 10 min, 20 min, 25 min) was varied for further investigations. Temperature rises were recorded by thermocouples (type K) attached to the front side of the specimens by a high-temperature resistant tape. The thermocouples were located close to the center of each panel.

A scanning electron microscope (SEM; SM-300, TOPCON Corp., Tokyo, Japan) was used for the determination of fiber diameters. For each given value, the diameters of at least 30 fibers are averaged. Energy-dispersive X-ray spectroscopy (EDX) analyses were done on an EVO HD 25 (Carl Zeiss Microscopy GmbH, Jena, Germany) scanning electron microscope.

Interlaminar shear strength (ILSS) tests were performed for 2 mm thick specimens in accordance to EN 2563 by a three-point flexural test with a universal mechanical testing machine (Z020, ZwickRoell GmbH & Co. KG, Ulm, Germany) [38].

## 3. Results and Discussion

### 3.1. Characterization of Phosphorus-Containing Polyacrylamides

The thermal stability of the newly synthesized polymeric flame retardants was determined by TG analysis. The temperature at maximum mass loss rate (T_max_), the initial degradation temperatures (T_1%_, T_5%_) at which 1% or 5% mass loss is reached and the residue at 800 °C are summarized in Table 2.

**Figure 2:**
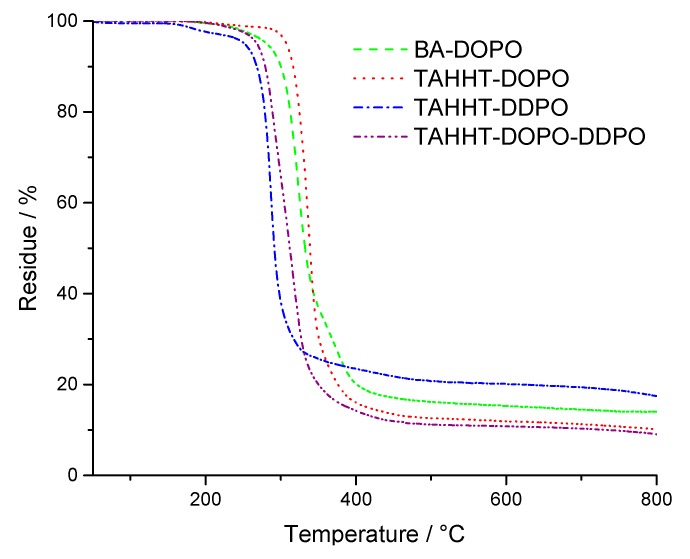
TG-curves of phosphorus containing polyacrylamides measured under N_2_-atmosphere.

**Figure 3:**
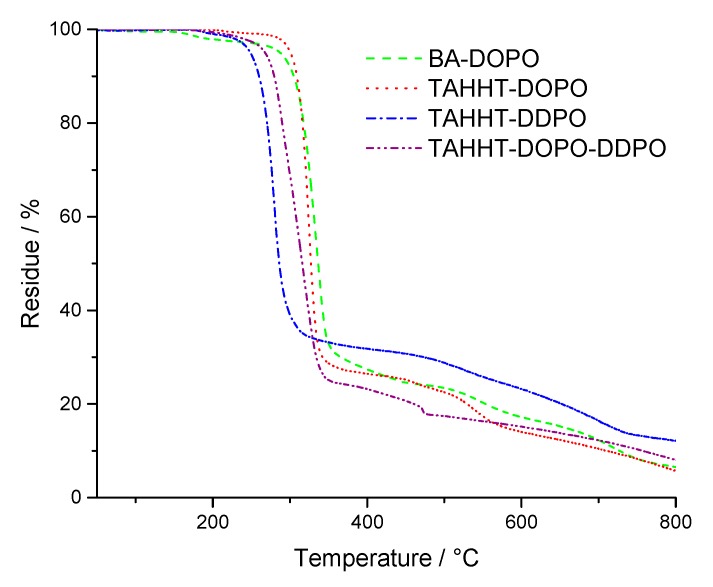
TG-curves of phosphorus containing polyacrylamides measured in air.

Figure 2 shows that DOPO-containing flame retardants like BA-DOPO and TAHHT-DOPO decompose at higher temperatures (T_5%,N2_ = 281 °C/307 °C) than DDPO-containing flame retardants like TAHHT-DDPO (T_5%,N2_ = 255 °C) under nitrogen atmosphere. The thermal stability of TAHHT-DOPO-DDPO is a combination of the thermal stabilities of TAHHT-DOPO and TAHHT-DDPO (T_5%,N2_ = 268 °C), as expected. The residue is highest for TAHHT-DDPO (19%). The results of measurements in synthetic air are shown in Figure 3. After similar initial degradation temperatures for measurements under nitrogen atmosphere and the char formation in the first step, this residue, that is highest for TAHHT-DDPO (33% at 419 °C), is decomposed slowly.

### 3.2. Neat Resin Samples and Carbon Fiber-Reinforced Composites

#### 3.2.1. Material Characterization

Glass transition temperatures were determined by dynamic mechanical analysis (DMA) as the maximum of the dissipation factor tan(δ): T_g_(Max(tan(δ))). The results are shown in Table 3. The incorporation of 10% phosphorus containing polyacrylamides leads to a maximum decrease of 13 °C, meaning a minor change of the glass transition temperature according to literature [1,24]. This change is caused by the low plasticizer effect of the oligomeric structures. 

Moisture uptake was determined by storing samples in water at 70 °C for 14 days. The increase in mass compared to the initial mass is 1.02% for the pure RTM6 and all other samples except for TAHHT-DOPO with 1.03%. According to this, the flame retardants have almost no influence on the moisture uptake. Glass transition temperatures of wetted samples (T_g,wet_) are similar to the temperatures of the original samples, because drying occurs before the glass temperature is reached. Slight decreases of the glass transition temperatures are observed since the hydrophilic phosphorous species prevent a complete drying in the given timespan. However, these changes are insignificant.

Table 4 presents interlaminar shear strength (ILSS) of the investigated CFRP samples. Values are additionally normalized to the CFRP without flame retardants. The incorporation of 10% polyacrylamides leads to no significant change of ILSS, which is within the measurement tolerance of the testing method (±10%). Therefore the application of the investigated flame retardants in epoxy based composites is possible with respect to mechanical performance.

#### 3.2.2. Thermal Properties

**Figure 4:**
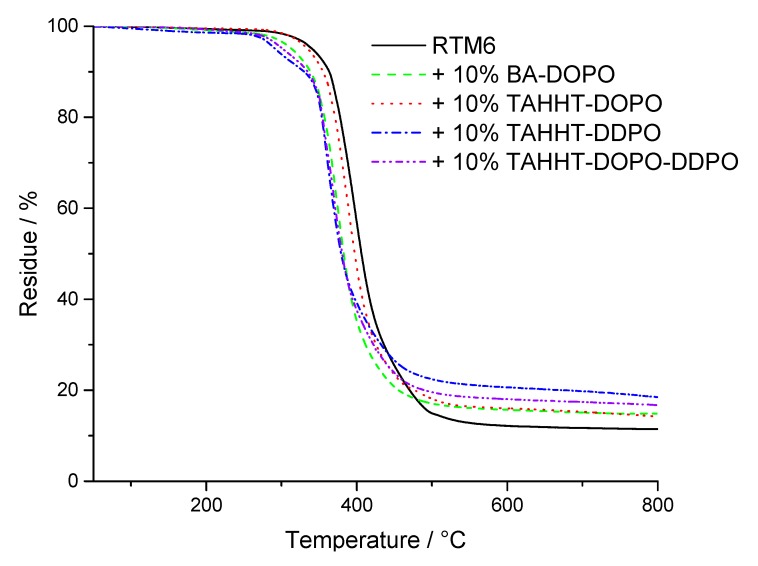
TG-curves of RTM6 and flame-retarded samples measured under N_2_-atmosphere.

**Figure 5:**
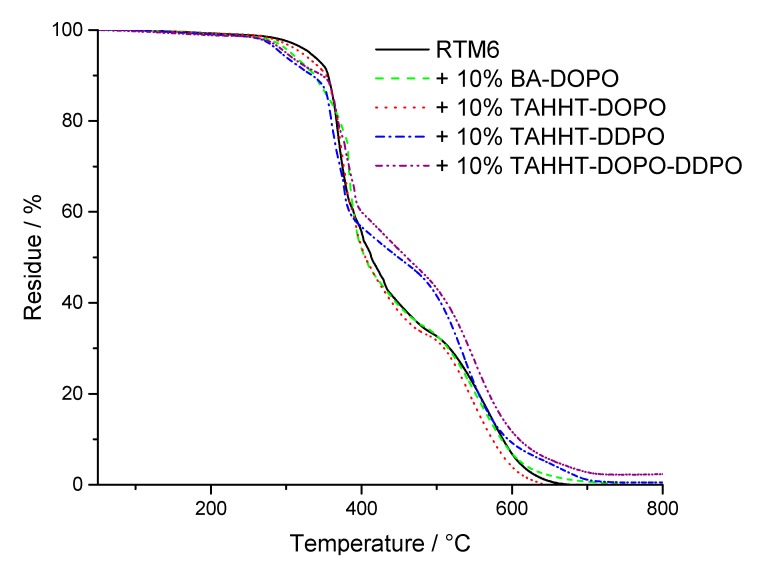
TG-curves of RTM6 and flame-retarded samples measured under air.

The thermal properties of the flame retardants and RTM6 formulations thereof were investigated by TGA under nitrogen atmosphere and in air. Figure 4 shows one step decomposition processes for RTM6 and flame retarded formulations under nitrogen atmosphere and Figure 5 shows two step decomposition processes in air. The temperature at maximum mass loss rate (T_max_), the initial degradation temperatures (T_1%_, T_5%_) at which 1% or 5% mass loss is reached and the residue at 800 °C are summarized in Table 5.

Under nitrogen atmosphere, the initial degradation temperatures T_1%_ and T_5%_ are lowered for every flame retardant, which indicates a flame-retarding action before the decomposition of the overall matrix. The residues are higher for flame retarded samples indicating the formation of char. It is highest for TAHHT-DDPO with 19%. The high residue shows a strong charring effect of TAHHT-DDPO in RTM6 resulting from the formation of polyphosphoric acid and the catalysis of the carbonification of the cured resin [39,40]. This charring layer is efficient to form a heat-barrier during combustion. RTM6 samples with other polyacrylamides added show higher residues than neat RTM6 as well. 

The simplified decomposition model by Rose and co-workers [41,42] assumes three steps for the decomposition of epoxy resins in air. An undistinctive first step is caused by dehydration leading to the formation of a moisture-free resin system. In a temperature range of 300 °C to 450 °C, the decomposition into stable char and different volatile species takes place. The third reaction taking place at temperatures above 450 °C is responsible for further degradation of the stable char. For RTM6 and DOPO-containing polyacrylamides, more volatiles are set free in the first decomposition step, leading to fewer residues before the second decomposition step occurs, suggesting gas-phase activity for these flame retardants.

#### 3.2.3. Flame-Retardant Performance

**Figure 6:**
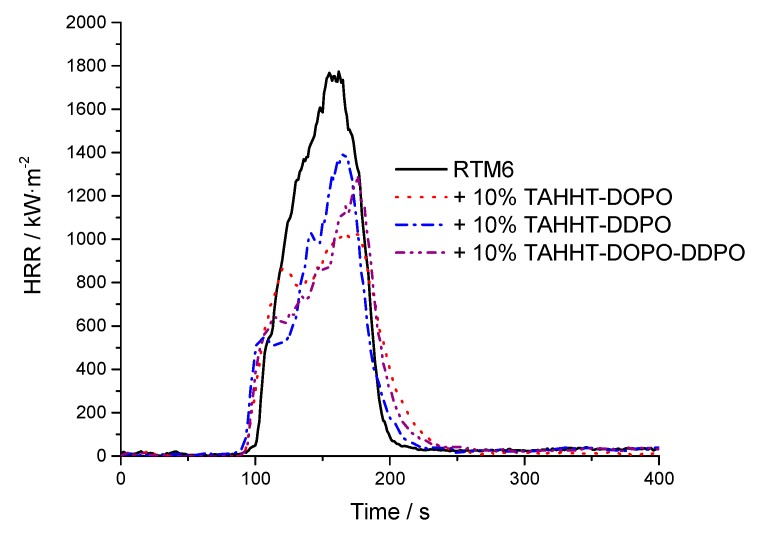
Heat release rate (HRR) of non-reinforced samples at 35 kW·m^−2^.

**Figure 7:**
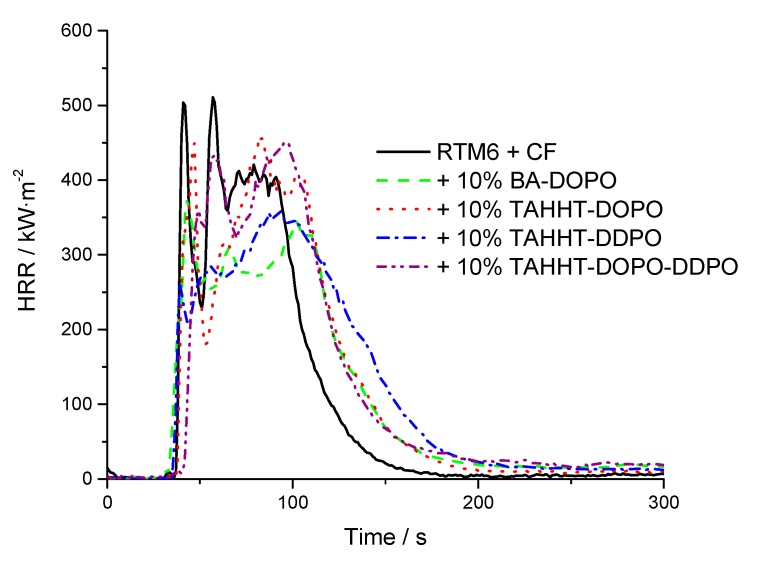
HRR of reinforced samples at 60 kW·m^−2^.

Fire tests of the neat resin (4 mm samples) under forced conditions were done at a heat flux of 35 kW·m^−2^ in a cone calorimeter. This heat flux is suitable for the investigation of the burning behavior. Composite materials (2 mm samples) were investigated at a heat flux of 60 kW·m^−2^ respectively to quantifiably observe the fiber degradation within 20 min. The HRR (heat release rate) curves vs. time of neat resins are presented in Figure 6 and the curves of the composites in Figure 7. Values of important parameters including time to ignition (tti), peak of heat release rate (pHRR), total heat release (THR), maximum average rate of heat emission (MARHE) and the total smoke release (TSR) values are summarized in Table 6. For the 2 mm reinforced samples the sum parameters were determined after 300 s of testing and for the 4 mm neat resin samples after 500 s. Hand lay-up leads to different fiber volume ratios, so the combustible material amount differs for the different reinforced samples, which is considered by the value X as the ratio of the combustible material mass (equal to the mass of the matrix) to the whole sample mass. In addition UL94-test classifications of 4 mm thick samples are specified in Table 6.

The burning behavior of neat RTM6 is characterized by a sharp peak in HRR, which is characteristic for a rapid and continuous combustion. For flame retarded samples the development of the HRR during the measurement shows a lower heat release shortly after burning, resulting in a peak at a comparable time to the non-flame retarded sample. The addition of flame retardants to RTM6 leads to decreasing pHRR-, THR- and MARHE-values. The reduction of pHRR is the highest for the sample flame retarded with TAHHT-DOPO by 36%. Literature [9] showed a comparable effect of other DOPO and DDPO-containing flame retardants in neat RTM6 samples. In particular the addition of TAHHT-DDPO leads to a decrease of the HRR just after ignition which is essential for the further burning process as the decrease of HRR indicates the formation of char [37]. The addition of TAHHT-DOPO delays the tti whereas DDPO-containing polyacrylamides decrease the tti. This observation is characteristic, because the gas phase activity of DOPO inhibits the ignition whereas a char is not formed before combustion.

UL94 measurements show that an addition of 10 wt% of flame retardants results in V0 classifications for all samples except for BA-DOPO. The fiber reinforcement acts as inert filler. Therefore, the RTM6-composite reaches V1 classification even without an additional flame retardant and V0 classification for flame-retarded samples. The fiber-reinforcement leads to significant differences in the HRR-curves in comparison to non-reinforced material. Delamination processes are represented in the curves by sharp peaks shortly after ignition. Single plies are detached from others and they burn fierce and fast due to gas between the fiber plies acting as a heat barrier. The reinforcement significantly reduces pHRR, THR, MARHE and TSR. The relative changes caused by the incorporation of flame retardants are comparable to the non-reinforced samples, but the high heat flux of 60 kW·m^−2^ required for fiber degradation leads to significant standard deviations, so that the detailed comparison of the different flame retardants is not possible.

The THR/ML (ML: mass loss) value gives reliable information on the flame retardant mechanism [37] as it stands for the total heat evolved per mass that is released in form of volatiles. The smaller this value is in comparison to the non-flame retarded sample (non-reinforced: 2.25 kW·m^−2^·g^−1^/reinforced: 2.51 kW·m^−2^·g^−1^), the more gas phase activity occurs during combustion. According to the value of THR/ML, TAHHT-DDPO acts mainly in the condensed phase (non-reinforced: 2.19 kW·m^−2^·g^−1^/reinforced: 2.37 kW·m^−2^·g^−1^) which is also shown by the low TSR·X^−1^ (3853 m^2^·m^−2^) of the non-reinforced sample. DOPO containing polyacrylamides show a distinctive gas phase activity.

The overall amount of formed smoke (TSR·X^−1^) is higher for flame retarded composite samples than for the non-flame retarded samples. 

**Figure 8:**
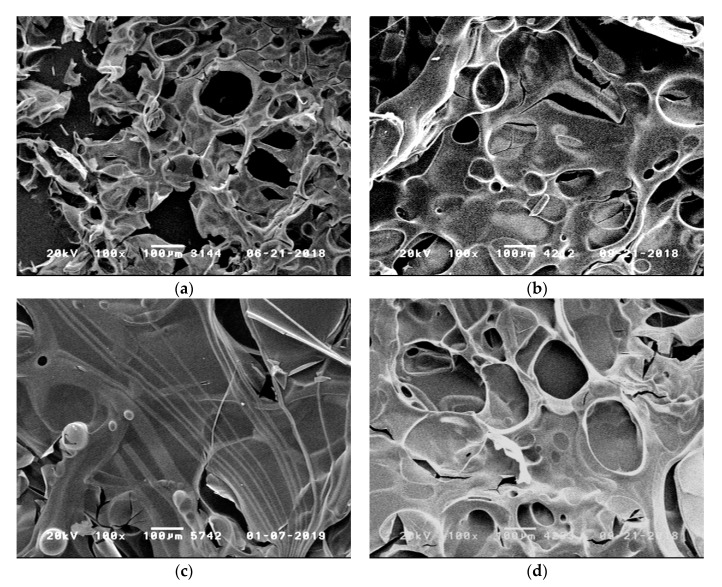
Scanning electron microscope (SEM) images of the residues obtained after cone calorimetry at 35 kW·m^−2^ for 10 min: (**a**) RTM6; (**b**) RTM6 + 10% TAHHT-DOPO; (**c**) RTM6 + 10% TAHHT-DDPO; (**d**) RTM6 + 10% TAHHT-DOPO-DDPO.

Figure 8 shows SEM images of the residues obtained after cone calorimetry at 35 kW·m^−2^ for 10 min. While RTM6 alone forms a highly instable residue that contains small holes (diameter about 50–100 µm), the addition of phosphorus containing flame retardants leads to residues that are more stable and covered with less holes. In particular, the addition of TAHHT-DDPO to RTM6 shows a continuous layer.

#### 3.2.4. Fiber Protection

SEM was used to determine mean diameters and smallest fiber diameters of carbon fibers for different samples shown in Table 7. The determined initial diameter of G0939 carbon fibers is (7.3 ± 0.3) µm. For composites not containing any flame retardant a mean fiber diameter of (3.3 ± 0.7) µm and a mentionable amount of respirable fiber fragments are observed after irradiation at 60 kW·m^−2^ (20 min). For each investigated flame retardant, no respirable fiber fragments were observed under the same conditions. The incorporation of TAHHT-DDPO showed the best fiber protection with a mean fiber diameter of (6.3 ± 0.4) µm after irradiation at 60 kW·m^−2^ (20 min). Flame retardants that contain only DOPO as phosphorus source, show slightly smaller mean fiber diameters (BA-DOPO: (5.2 ± 0.3) µm, TAHHT-DOPO: (5.3 ± 0.2) µm). The different substituted polyacrylamide TAHHT-DOPO-DDPO shows a mean fiber diameter of (5.9 ± 0.3) µm after irradiation that is in between the mean diameters of the flame retardants with only one phosphorus source, as expected.

**Figure 9:**
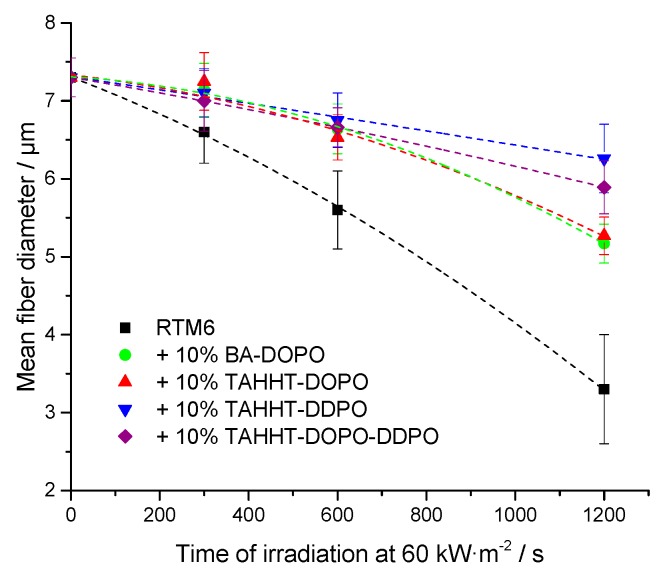
Mean fiber diameter in dependence on irradiation time at 60 kW·m^−2^ and flame retarded matrix composition.

**Figure 10:**
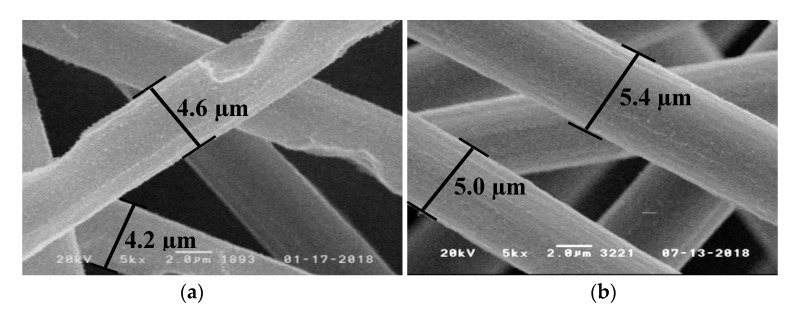
SEM images of carbon fibers after irradiation at 60 kW·m^−2^: **a**: RTM6 + CF, 1200 s; **b**: RTM6 + 10% TAHHT-DOPO-DDPO, 1500 s.

Figure 9 shows the mean fiber diameter depending on irradiation time at 60 kW·m^−2^ for different resin formulations. Carbon fibers in RTM6 without addition of flame retardants show a steady decrease in diameter. After 5 min a mean diameter of (6.6 ± 0.4) µm is reached, after 20 min (3.3 ± 0.7) µm. Within the observed irradiation time of 20 min, the fiber degradation is retarded sufficiently by addition of any phosphorus containing polyacrylamide. After 300 s no significant degradation of the fiber diameter occurs. The results for BA-DOPO and TAHHT-DOPO show similar curve progressions. TAHHT-DDPO is the most effective flame retardant for fiber protection. The curve for TAHHT-DOPO-DDPO containing samples is in between, as expected. An additional composite containing 10% TAHHT-DOPO-DDPO that was irradiated for 1500 s still exhibits a mean diameter of (5.4 ± 0.3) µm. Figure 10 shows SEM images of carbon fibers after irradiation at 60 kW·m^−2^. While the composite which does not contain any flame retardants (Figure 10a) tends to form holes at the fiber surface within 1200 s, the sample with 10% TAHHT-DOPO-DDPO shows no defects after 1500 s (Figure 10b).

**Figure 11:**
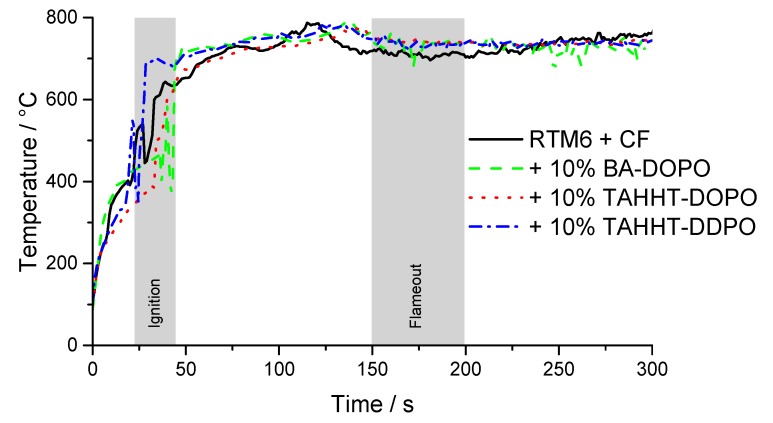
Temperature at the sample surface during irradiation (60 kW·m^−2^) measured by a thermocouple attached to the surface.

In order to determine the mechanism of fiber protection, the temperature on the irradiated surface during combustion was measured by a thermocouple attached to the sample. The results are shown in Figure 11. Temperatures above 600 °C necessary for fiber degradation [15] are reached during the combustion of the matrix resin. There are no distinct differences in the shape of these curves suggesting that there is no influence of the surface temperature on the resulting mean fiber diameter, but the added flame retardants are responsible for fiber protection.

The results of EDX measurements are summarized in Table 8. After 1200 s at 60 kW·m^−2^, the surface of carbon fibers in the non-flame retarded composite sample consists of 94.3% carbon, 4.2% nitrogen and 1.4% oxygen. Interestingly, DDPO-containing samples show higher P- and O-contents on the fiber surface after irradiation. The incorporation of TAHHT-DOPO leads to 0.7% P and 2.8% O on the fiber surface after irradiation. Whereas TAHHT-DDPO containing samples showed 2.4% P and 8.8% O and TAHHT-DOPO-DDPO containing samples showed 1.4% P and 6.8% O on the fiber surface after irradiation. This corresponds to the predicted activity in the condensed phase for DDPO by the formation of polyphosphoric acid. However, with prolonged irradiation polyphosphoric acid is degraded and fiber protection decreases. During the irradiation under the cone heater a loose matrix residue is obtained on top of the sample and between the laminate plies that is decreased constantly during the run. 

### 3.3. Synergistic Mixtures with Boehmite

#### 3.3.1. Thermal Properties

The thermal properties of RTM6 formulations with boehmite and the phosphorus-containing flame retardants TAHHT-DDPO and TAHHT-DOPO were determined by TG measurements. Two types of boehmite were tested: synthetic boehmite Actilox^®^ B30 (Actilox) and Disperal^®^ 40 (Disperal). Actilox^®^ B30 is a high purity boehmite (99%) with a particle size D_50_ of 1.8 µm and specific surface area (BET) of 3 m²·g^−1^. Disperal^®^ 40 is an acid dispersable boehmite with lower purity (94%) and bigger particles (35 µm) with higher BET-surface (100 m²·g^−1^) [43,44].

**Figure 12:**
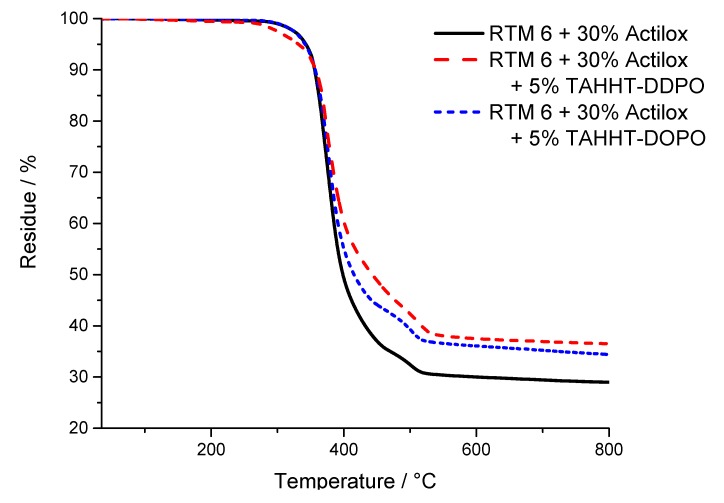
TG curves of Actilox-containing samples measured under N_2_-atmosphere.

**Figure 13:**
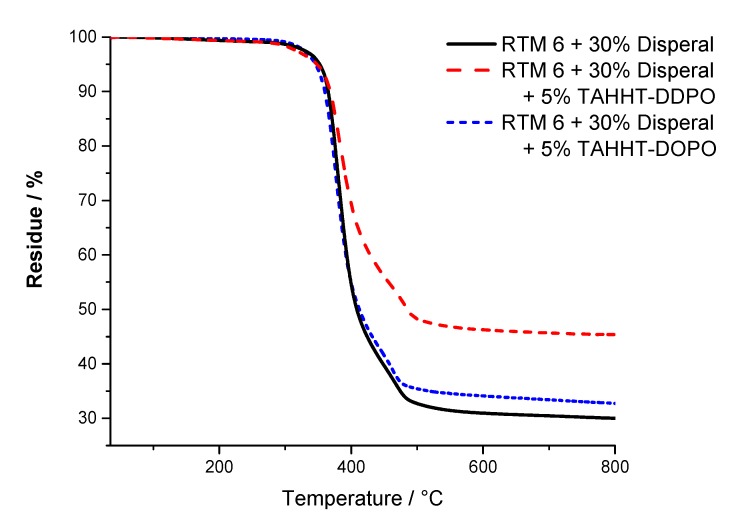
TG curves of Disperal-containing samples measured under N_2_-atmosphere.

The results for the temperature at maximum mass loss rate (T_max_), the initial degradation temperatures (T_1%_, T_5%_) at which 1% or 5% mass loss is reached and the residue at 800 °C are summarized in Table 9. Measurements under nitrogen atmosphere showed similar results for samples containing Actilox (Figure 12) and Disperal (Figure 13) in terms of temperatures at 5% mass lost and residue. In combination with TAHHT-DOPO the temperatures with 1% and 5% mass lost are not affected significantly for Actilox-containing samples. The temperature with 1% mass lost is increased by 20 °C to 305 °C for Disperal-containing samples. The residue at 800 °C is increased by 5% with Actilox and 2% with Disperal. The temperature at 1% mass lost is decreased by more than 20 °C for both boehmite types with TAHHT-DDPO. On the other hand, the temperature at 5% mass lost is just decreased slightly by TAHHT-DDPO with both types of boehmite. With this phosphorus containing compound, the residue is significantly higher, leading to 46% residue at 800 °C for Disperal + TAHHT-DDPO.

**Figure 14:**
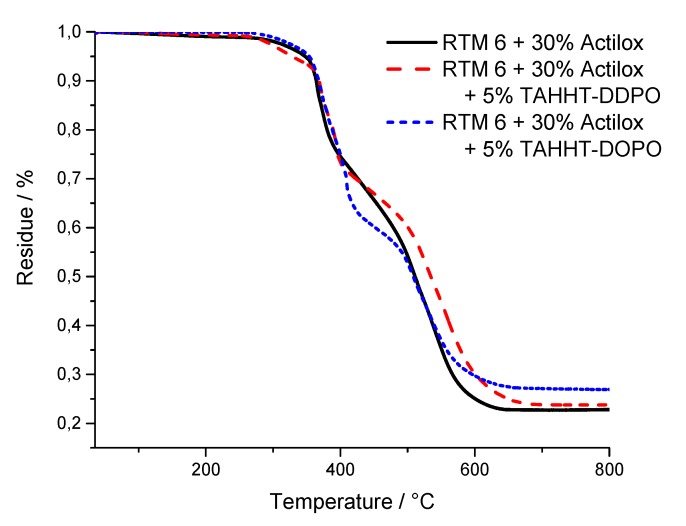
TG curves of Actilox-containing samples measured under air.

**Figure 15:**
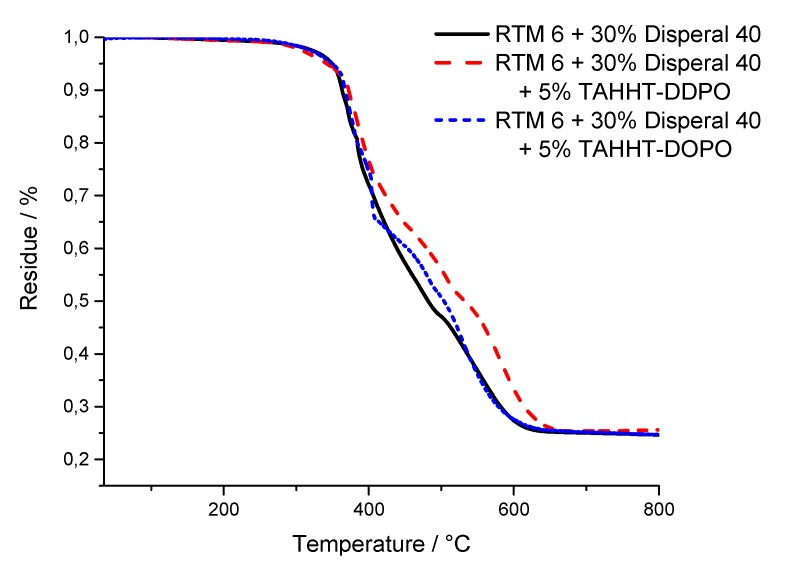
TG curves of Disperal-containing samples measured under air.

TG measurements under synthetic air show similar results to measurements under nitrogen atmosphere. There is no significant difference between samples containing Actilox or Disperal except for RTM 6 + 30% Actilox. Its temperature at 1% mass lost is 52 °C lower than for the equivalent Disperal-containing sample. The incorporation of any phosphorus containing polyacrylamide leads to higher temperatures at 1% mass lost and lower temperatures at 5% mass lost. The residues at 800 °C are higher as well, but lower than under nitrogen. The graphs show just one decomposing step under nitrogen, but two steps under synthetic air (Figure 14 and Figure 15). The first step is correlated to char formation. The second step, which is more distinctive for Actilox-containing samples, is correlated to char decomposition and formation of more volatiles. The reaction of boehmite to aluminum oxide occurs at similar temperatures like the decomposition of char [41,42]. This step occurs for all formulations between 510 and 530 °C. The temperatures of highest mass lost rate for the first maximum are about 370 °C and not affected significantly by the different flame retardants as well as the second maximum between 530 and 540 °C. The formulations with TAHHT-DDPO have a higher last T_max_, leading to the highest T_max_ at 581 °C for Disperal + TAHHT-DDPO. For the samples containing synergists, there is another peak around 400 °C. These peaks do not differ for the different formulations except for Disperal + TAHHT-DDPO with a missing peak. This step overlaps with the step of char formation.

For comparing purposes, the residue at the inflection point is examined. This point differs in residue and temperature between Actilox- and Disperal-containing specimens. On the one hand for Actilox-containing samples, the temperatures do not differ significantly. The residue is for TAHHT-DDPO slightly higher and for TAHHT-DOPO lower. On the other hand for Disperal-containing samples, the residue is constantly about 61%. But the temperature of the inflection point rises with synergists. Therefore the combination of TAHHT-DDPO and Disperal in RTM6 causes the highest temperature at inflection point with 474 °C.

#### 3.3.2. Flame-Retardant Performance

**Figure 16:**
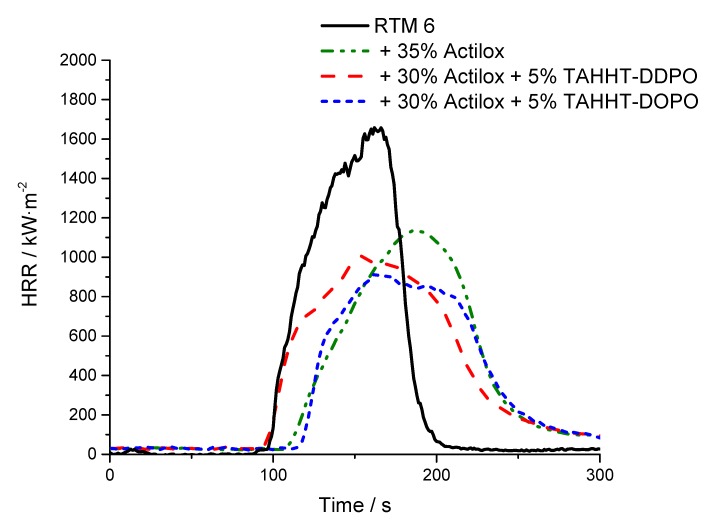
HRR of different specimen with and without Actilox B30 at 35 kW·m^−2^.

**Figure 17:**
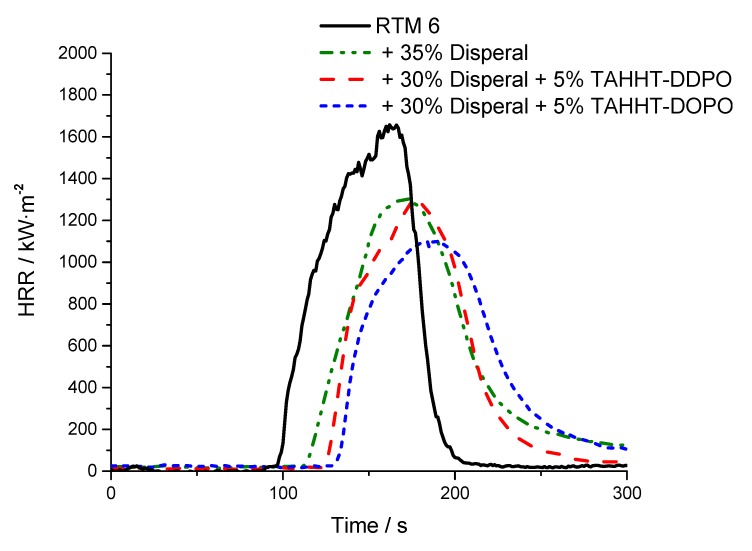
HRR of different specimen with and without Disperal 40 at 35 kW·m^−2^.

In order to determine the flame-retardant performance of different samples, UL94-tests and cone calorimetry measurements were performed. The results are summarized in Table 10. HRR curves are shown in Figure 16 and Figure 17. In UL94 tests all specimens containing Disperal 40 reach no classification. Only a combination of Actilox B30 and TAHHT-DDPO in RTM6 achieves V0 rating. Results from cone calorimetry (35 kW·m^−2^, 500 s) showed a delay of tti by 10 to 20 s for both boehmite types. The residue is increased by adding a synergist to Disperal-containing formulations. The residue of the specimen containing Actilox is not affected significantly by the incorporation of phosphorus-containing acrylamides. Other important parameters like pHRR, THR, MARHE and TSR are decreased. pHRR is decreased by boehmite additives, but also decreased by the addition of phosphorus containing polyacrylamides. It drops by 34% using Actilox B30 and by 22% with Disperal 40. Both synergists lower the pHRR with the formulation of TAHHT-DOPO and Actilox B30 in RTM6 having the lowest result. The pHRR is decreased by 46% to 919 kW m². The additives affect not only the peak, but the whole curve (Figure 16). Whilst pure RTM6 shows a peak in HRR curve in the end of its burning, adding boehmite leads to broader, smaller peaks with maximum in the center.

The THR/ML value gives reliable information on the flame-retardant mechanism [37] as it stands for the total heat evolved per mass that is released in form of volatiles. A decrease by 18% is observed using TAHHT-DOPO and boehmite in comparison to pure RTM 6. This indicates more gas-phase activity which can be conferred to an increase of TSR The addition of TAHHT-DOPO leads to a small increase of TSR by 5% for Actilox B30, but it increases by 24% for Disperal 40. THR/ML and TSR do not distinguish significantly between the boehmite types in combination with each synergist. MARHE shows the same tendency. It is decreased by boehmite as well as by the synergists. It is affected most by the incorporation of TAHHT-DOPO leading to a decrease by 35% with Actilox B30 conferred to pure RTM 6.

**Figure 18:**
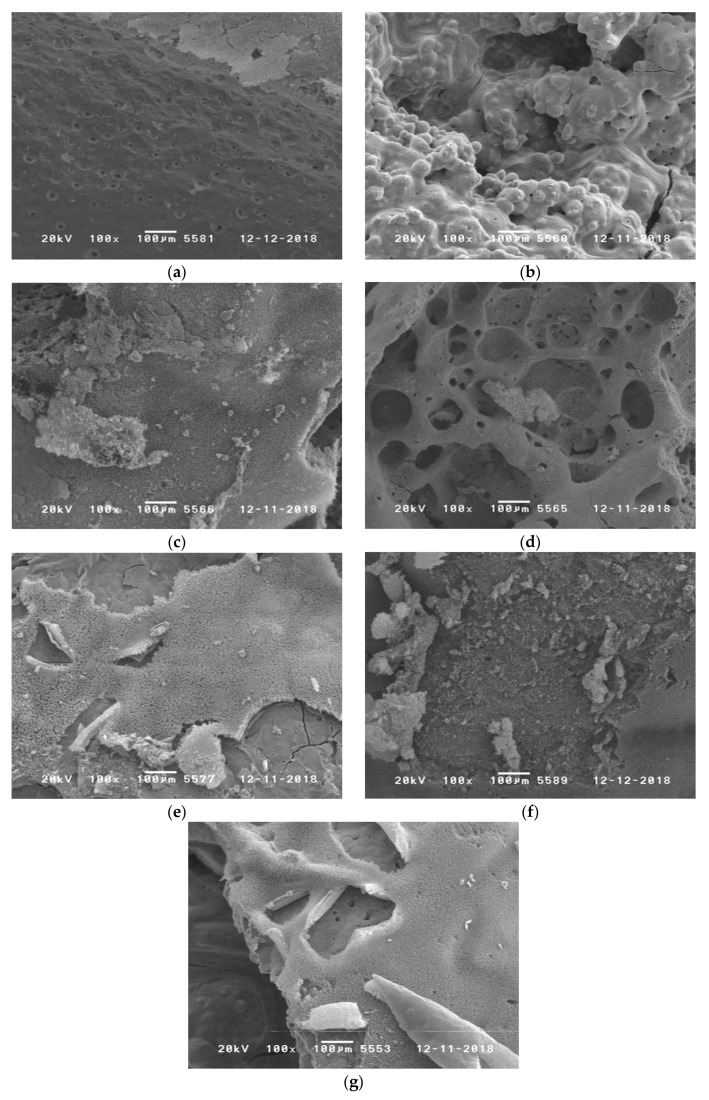
SEM images of the residues obtained after cone calorimetry for RTM6 samples at 35 kW·m^−2^ for 500 s with: (**a**) 35% Actilox; (**b**) 30% Actilox + 5 % TAHHT-DOPO; (**c**) 30% Actilox + 5% TAHHT-DDPO surface; (**d**) 30% Actilox + 5% TAHHT-DDPO inside a hole; (**e**) 35% Dispersal; (**f**) 30% Dispersal + 5% TAHHT-DOPO; (**g**) 30% Dispersal + 5% TAHHT-DDPO.

Figure 18 shows SEM images of the residues obtained after cone calorimetry at 35 kW·m^−2^ for 500 s. The addition of Actilox B30 to RTM6 leads to a non-continuous char surface with fractures indicating a fragile surface. Holes remain suggesting the possibility of oxygen and heat exchange. Voluminous structures were obtained with TAHHT-DOPO and Actilox B30 in RTM6. Specimens containing TAHHT-DDPO show a rough surface with a network-like structure underneath. Disperal 40 leads to a small layer of rough surface without holes. In some spots, the surface is broken and shows little holes underneath like the samples with Actilox B30. This surface is nearly completely decomposed for TAHHT-DOPO indicating a high gas-phase activity of this flame retardant. Voluminous structures are not observed with Disperal 40. 

The pictures show that during combustion a brittle char is developed. Only for V0 rating, a firm char layer is needed in order to inhibit oxygen and gas exchange [31]. The sample with Actilox and TAHHT-DDPO (Figure 18c) shows a continuous char surface correlating to V0 rating in UL94. Particularly for the Actilox and TAHHT-DOPO containing sample (Figure 18b), spherical particles with a diameter of about 50 µm can be observed. The residue of small Actilox particles (1.8 µm diameter) acts as initial nuclei for crystallization. This is also observed in the TG measurements under synthetic air discussed in Section 3.3.1 by the second decomposition step that is more distinctive for Actilox than for Disperal (35 µm diameter). This is an additional flame-retardant activity in the condensed phase at temperatures above 400 °C, but the amount of phosphorus-containing synergist is not sufficient for a UL94 V0 rating. Literature [31] showed for ATH in unsaturated polyester resins that crystalline particles with a smaller specific surface area and phosphorus species with a hydrogen/carbon rich environment show gas-phase activity as well as the formation of a dense and tough residue, whereas the use of amorphous particles with a bigger specific surface area formed a fragile residue. This observation corresponds to this work, as Actilox has a BET-surface of 3 m²·g^−1^ and Disperal a BET surface of 100 m²·g^−1^.

## 4. Conclusions

The successful synthesis of novel phosphorus-containing polyacrylamides allows different applications. The synthesis itself is simple, versatile, and combines phosphorus and nitrogen in polymeric flame retardants. The selection of phosphorus species and multifunctional acrylamides as well as the ratio of the components is open. Different product properties like thermal stability or predominant flame retardant mechanism can be designed. Thermoset or thermoplastic polymers can be synthesized. In particular, the thermoplastics are soluble in epoxy resins and suitable for injection-molding processes. 

This research deals with the polymeric phosphorus containing flame retardants BA-DOPO, TAHHT-DOPO, TAHHT-DDPO and TAHHT-DOPO-DDPO. The thermal stability determined by TG measurements showed that DOPO combined with BA is decomposed at lower temperatures than DOPO combined with TAHHT. The flame-retardant efficiency was tested in neat RTM6 resin and in carbon fiber reinforced composite materials. A second focus was the retardation of the fiber degradation leading to respirable fiber fragments with diameters below 3 µm. While the gas phase active DOPO containing flame retardants showed the best flame retardant efficiency by decreasing pHRR, THR and MARHE and delaying tti in cone calorimetric measurements, TAHHT-DDPO showed the best fiber protection caused by the observed flame retardant activity in the condensed phase (char formation). In agreement with the fiber protection and flame retardancy, the mixed polyacrylamide TAHHT-DOPO-DDPO is the most efficient. In conclusion, by incorporation of any investigated phosphorus-containing flame retardant in amounts of 10% by matrix weight, the fiber degradation can be retarded sufficiently as no respirable fiber fragments and no formation of defects on the fiber surface were observed by SEM. No relation of surface temperature during combustion and fiber degradation was found. The effect of fiber protection is a result of a loose residue on the fibers and between the laminates as shown by EDX measurements. Since the polarity of the formed phosphate does not fit carbon fibers, the aim to create a continuous protection layer on the fiber surface still has to be pursued. The use of the flame-retarded RTM6 in high-performance composites is still possible since interlaminar shear strength, T_g_ and hot-wet properties were not affected significantly by the incorporation of these flame retardants.

Additional investigations with boehmite containing RTM6 samples showed the possible use of the phosphorus containing polyacrylamides as synergist with inorganic fillers. Especially the formulation of RTM6 with TAHHT-DDPO and Actilox B30 showed a continuous residue surface which explains the V0 classification in UL94 tests. The particles of Actilox B30 have a smaller specific surface area than the particles of Disperal 40 and act as nuclei for the formation of a continuous char layer, providing an additional activity in the condensed phase at temperatures above 400 °C. In particular, the residue of the RTM6 formulation with Actilox B30 and TAHHT-DOPO showed spherical particles after combustion.

## Figures and Tables

**Table 1:** Prepared samples based on RTM6 resin.

Flame Retardant in RTM6-Matrix	Reinforcement	Thickness/mm
**-**	-	4
10% TAHHT-DOPO	-	4
10% TAHHT-DDPO	-	4
10% TAHHT-DOPO-DDPO	-	4
-	CF	2
10% BA-DOPO	CF	2
10% TAHHT-DOPO	CF	2
10% TAHHT-DDPO	CF	2
10% TAHHT-DOPO-DDPO	CF	2
35% Actilox B30	-	4
30% Actilox B30 + 5% TAHHT-DDPO	-	4
30% Actilox B30 + 5% TAHHT-DOPO	-	4
35% Disperal 40	-	4
30% Disperal 40 + 5% TAHHT-DDPO	-	4
30% Disperal 40 + 5% TAHHT-DOPO	-	4

CF: carbon fiber.

**Table 2:** T_max_, T_1%_, T_5%_ and residue of phosphorus containing polyacrylamides obtained by thermogravimetric (TG) measurements in air or under nitrogen atmosphere with a heating rate of 10 K·min^−1^.

Sample	T_max_/°C	T_1%_/°C	T_5%_/°C	Residue/%
BA-DOPO, N_2_	323	220	281	15 (800 °C)
TAHHT-DOPO	337	231	307	10 (800 °C)
TAHHT-DDPO	287	181	255	19 (800 °C)
TAHHT-DOPO-DDPO	315	220	268	9 (800 °C)
BA-DOPO, air	I 335 | II 549 | III 717	178	290	25 (453 °C) | 7 (800 °C)
TAHHT-DOPO	I 237 | II 300	237	300	25 (544 °C) | 6 (800 °C)
TAHHT-DDPO	I 280 | II 526 | III 699	209	250	33 (419 °C) | 14 (800 °C)
TAHHT-DOPO-DDPO	I 317 | II 472	213	268	22 (420 °C) | 8 (800 °C)

**Table 3:** Glass transition temperatures and moisture uptake of RTM6 samples.

Sample	T_g_(Max(tan(δ))) ^1^/°C	Moisture uptake/%	T_g,wet_(Max(tan(δ))) ^1^/°C
**RTM6**	215	1.02	218
**+ 10% BA-DOPO**	208	1.02	205
**+ 10% TAHHT-DOPO**	205	1.02	202
**+ 10% TAHHT-DDPO**	202	1.03	198
**+ 10% TAHHT-DOPO-DDPO**	204	1.02	197

^1^ Error in measurement ±3 °C.

**Table 4:** Interlaminar shear strength (ILSS) of selected carbon fiber-reinforced thermoset polymers (CFRP) samples additionally normalized to RTM6 + CF.

Sample	ILS (N·mm^−2^)	Relative ILSS (%)
RTM6 + CF	67.8 ± 3.2	100 ± 5
+ 10% BA-DOPO	65.4 ± 2.0	97 ± 3
+ 10% TAHHT-DOPO	67.4 ± 1.8	99 ± 3
+ 10% TAHHT-DDPO	71.0 ± 2.3	105 ± 4

**Table 5:** T_max_, T_1%_, T_5%_ and residue of formulations of RTM6 and phosphorus containing flame retardants obtained by TG measurements under air or nitrogen atmosphere with a heating rate of 10 K·min^−1^.

Sample	T_max_/°C	T_1%_/°C	T_5%_/°C	Residue/%
RTM6, N_2_	379	265	341	10 (800 °C)
+ 10% BA-DOPO	373	211	314	15 (800 °C)
+ 10% TAHHT-DOPO	389	284	335	14 (800 °C)
+ 10% TAHHT-DDPO	362	170	293	19 (800 °C)
+ 10% TAHHT-DOPO-DDPO	371	216	301	17 (800 °C)
RTM6, air	I 369 | II 572	229	331	33 (492 °C) | 0 (800 °C)
+ 10% BA-DOPO	I 384 | II 555	198	305	34 (488 °C) | 0 (800 °C)
+ 10% TAHHT-DOPO	I 373 | II 546	212	318	34 (479 °C) | 0 (800 °C)
+ 10% TAHHT-DDPO	I 360 | II 376 | III 537	198	293	52 (433 °C) | 1 (800 °C)
+ 10% TAHHT-DOPO-DDPO	I 301 | II 368 | III 550	190	301	51 (454 °C) | 3 (800 °C)

**Table 6:** Results of cone calorimetry (neat resin: sample thickness 4 mm, composites: sample thickness 2 mm) and UL94 (sample thickness 4 mm) for depicted samples.

Sample	tti/s	pHRR/kW·m^−2^	THR·X^−1^/MJ·m^−2^	MARHE/kW·m^−2^	TSR·X^−1^/m^2^·m^−2^	THR·ML^−11^/kW·m^−2^·g^−1^	Residue/%	X	UL94-V *
**RTM6**	94 ± 1	1715 ± 58	104 ± 1	550 ± 6	5601 ± 31	2.25	4 ± 2	1	NR
**+ 10% BA-DOPO**	-	-	-	-	-	-	-	1	V1
**+ 10% TAHHT-DOPO**	98 ± 12	1092 ± 54	89 ± 2	401 ± 22	6458 ± 51	1.85	2 ± 0	1	V0
**+ 10% TAHHT-DDPO**	81 ± 2	1419 ±8	85 ± 2	421 ± 3	3853 ± 17	2.19	3 ± 0	1	V0
**+ 10% TAHHT-DOPO-DDPO**	85 ± 1	1278 ± 30	85 ± 3	416 ± 8	5160 ± 123	1.97	2 ± 0	1	V0
**RTM6 + CF**	31 ± 2	492 ± 19	72 ± 1	242 ± 5	3763 ± 165	2.51	59 ± 1	0.43	V1
**+ 10% BA-DOPO**	32 ± 5	384 ± 53	63 ± 3	203 ±16	5815 ± 600	2.05	56 ± 1	0.49	V0
**+ 10% TAHHT-DOPO**	34 ± 1	476 ± 75	70 ± 3	234 ± 115	5070 ± 283	2.17	54 ± 1	0.52	V0
**+ 10% TAHHT-DDPO**	29 ± 4	388 ± 52	67 ± 4	218 ± 22	4277 ± 369	2.37	59 ± 1	0.50	V0
**+ 10% TAHHT-DOPO-DDPO**	37 ± 3	456 ± 58	69 ± 2	228 ± 13	5269 ± 136	2.21	55 ± 1	0.52	V0

X: ratio of the combustible material mass to the whole sample mass. * NR: not rated.

**Table 7:** Mean diameter, standard deviation and smallest measured diameter of at least 30 carbon fibers in composite materials with partially flame retarded RTM6 after irradiation at 60 kW·m^−2^ for 20 min determined by SEM.

Sample	Mean Diameter (µm)	Smallest Diameter (µm)
RTM6 + CF	3.3 ± 0.7	2.1
+ 10% BA-DOPO	5.2 ± 0.3	4.7
+ 10% TAHHT-DOPO	5.3 ± 0.2	4.9
+ 10% TAHHT-DDPO	6.3 ± 0.4	5.3
+ 10% TAHHT-DOPO-DDPO	5.9 ± 0.3	5.3

**Table 8:** Concentrations of elements at the fiber surface after irradiation.

Sample	C on surface/%	N on surface/%	O on surface/%	P on surface/%
RTM6 + CF 1200 s at 60 kW·m^−2^	94.3	4.2	1.4	not detected
+ 10% TAHHT-DOPO 1200 s at 60 kW·m^−2^	91.9	4.6	2.8	0.7
+ 10% TAHHT-DDPO 1200 s at 60 kW·m^−2^	84.0	4.3	8.8	2.4
+ 10% TAHHT-DOPO-DDPO 1200 s at 60 kW·m^−2^	86.3	4.9	6.8	1.4

**Table 9:** T_max_, T_1%_, T_5%_ and residue of boehmite and phosphorus containing polyacrylamides in RTM6 obtained by TG measurements under nitrogen atmosphere or synthetic air with a heating rate of 10 K·min^−1^.

Sample	T_max_/°C	T_1%_/°C	T_5%_/°C	Residue/%
RTM6, N_2_	379	265	341	10 (800 °C)
+ 30% Actilox	376	301	343	29 (800 °C)
+ 30% Actilox + 5% TAHHT-DDPO	373	274	333	37 (800 °C)
+ 30% Actilox + 5% TAHHT-DOPO	379	304	342	34 (800 °C)
+ 30% Disperal	374	285	353	30 (800 °C)
+ 30% Disperal + 5% TAHHT-DDPO	380	277	349	46 (800 °C)
+ 30% Disperal + 5% TAHHT-DOPO	376	305	346	33 (800 °C)
RTM6, air	I 369 | II 572	229	331	33 (492 °C) | 0 (800 °C)
+ 30% Actilox	I 366 | II 538	215	348	64 (458 °C) | 23 (800 °C)
+ 30% Actilox + 5% TAHHT-DDPO	I 371 | II 394 | III 558	267	329	66 (453 °C) | 24 (800 °C)
+ 30% Actilox + 5% TAHHT-DOPO	I 371 | II 409 | III 534	299	354	60 (456 °C) | 27 (800 °C)
+ 30% Disperal	I 386 | II 543	267	350	60 (439 °C) | 25 (800 °C)
+ 30% Disperal + 5% TAHHT-DDPO	I 383 | II 581	267	343	61 (474 °C) | 26 (800 °C)
+ 30% Disperal 40 + 5% TAHHT-DOPO	I 376 | II 404 | III 530	295	353	61 (445 °C) | 25 (800 °C)

* NR: not rated.

**Table 10:** Results of cone calorimetry and UL94 (sample thickness 4 mm) for depicted samples containing boehmite.

Sample	tti/s	pHRR/kW·m^−2^	THR/MJ·m^−2^	MARHE/kW·m^−2^	TSR/m^2^·m^−2^	THR·ML^−1^/kW·m^−2^·g^−1^	Residue/%	UL94-V *
RTM 6	94 ± 2	1715 ± 58	113 ± 2	550 ± 6	6305 ± 394	2.39	1.9 ± 1.4	NR
+ 35% Actilox B30	109 ± 12	1142 ± 14	107 ± 3	420 ± 13	5079 ± 94	2.38	5.7 ± 1.0	NR
+ 30% Actilox B30 + 5% TAHHT-DDPO	96 ± 5	1017 ± 60	99 ± 2	410 ± 1	4868 ± 118	2.24	33.6 ± 0.5	V0
+ 30% Actilox B30 + 5% TAHHT-DOPO	110 ± 4	919 ± 20	91 ± 3	359 ± 11	5309 ± 148	1.99	32.5 ± 0.7	NR
+ 35% Disperal 40	95 ± 7	1330 ± 22	102 ± 3	454 ± 22	4239 ± 249	2.11	25.8 ± 6.4	NR
+ 30 % Disperal 40 + 5% TAHHT-DDPO	108 ± 2	1289 ± 31	96 ± 3	423 ± 5	4511 ± 204	2.22	32.0 ± 0.7	NR
+ 30% Disperal 40 + 5% TAHHT-DOPO	117 ± 4	1065 ± 5	89 ± 4	372 ± 20	5261 ± 16	1.94	32.6 ± 1.1	NR

* NR: not rated.
